# Cost-effectiveness of reducing salt intake in the Pacific Islands: protocol for a before and after intervention study

**DOI:** 10.1186/1471-2458-14-107

**Published:** 2014-02-04

**Authors:** Jacqui Webster, Wendy Snowdon, Marj Moodie, Satu Viali, Jimaima Schultz, Colin Bell, Mary-Anne Land, Shauna Downs, Anthea Christoforou, Elizabeth Dunford, Federica Barzi, Mark Woodward, Bruce Neal

**Affiliations:** 1 George Institute for Global Health, (affiliated with the University of Sydney), Level 10, King George V Building, Royal Prince Alfred Hospital, Camperdown, Sydney, New South Wales 2050, Australia; 2 Pacific Research Centre for the Prevention of Obesity and Non-Communicable Diseases, Suva, Fiji; 3 College of Medicine, Nursing and Health Sciences, Fiji National University, Suva, Fiji; 4 Deakin Health Economics, Deakin University, Melbourne, Australia; 5 Medical Specialist Clinic and Ministry of Health, Apia, Samoa; 6 National Food and Nutrition Centre, Suva, Fiji; 7 School of Medicine, Deakin University, Melbourne, Australia; 8 Royal Prince Alfred Hospital, Sydney, Australia

**Keywords:** Salt, Sodium, Hypertension, Intervention, Surveillance, Diet, Food, Monitoring, Non-communicable diseases, Pacific Islands

## Abstract

**Background:**

There is broad consensus that diets high in salt are bad for health and that reducing salt intake is a cost-effective strategy for preventing chronic diseases. The World Health Organization has been supporting the development of salt reduction strategies in the Pacific Islands where salt intakes are thought to be high. However, there are no accurate measures of salt intake in these countries. The aims of this project are to establish baseline levels of salt intake in two Pacific Island countries, implement multi-pronged, cross-sectoral salt reduction programs in both, and determine the effects and cost-effectiveness of the intervention strategies.

**Methods/Design:**

Intervention effectiveness will be assessed from cross-sectional surveys before and after population-based salt reduction interventions in Fiji and Samoa. Baseline surveys began in July 2012 and follow-up surveys will be completed by July 2015 after a 2-year intervention period.

A three-stage stratified cluster random sampling strategy will be used for the population surveys, building on existing government surveys in each country. Data on salt intake, salt levels in foods and sources of dietary salt measured at baseline will be combined with an in-depth qualitative analysis of stakeholder views to develop and implement targeted interventions to reduce salt intake.

**Discussion:**

Salt reduction is a global priority and all Member States of the World Health Organization have agreed on a target to reduce salt intake by 30% by 2025, as part of the global action plan to reduce the burden of non-communicable diseases. The study described by this protocol will be the first to provide a robust assessment of salt intake and the impact of salt reduction interventions in the Pacific Islands. As such, it will inform the development of strategies for other Pacific Island countries and comparable low and middle-income settings around the world.

## Background

Non-communicable diseases (NCDs), including cardiovascular disease, cancer, diabetes and chronic respiratory disease are the leading cause of death in the world, killing more people each year than all other causes combined [1]. New Global Burden of Disease data indicate that one in four deaths worldwide were due to ischemic heart disease or stroke in 2010 [2] and that blood pressure is the most important global risk factor for health [3]. In response, the United Nations has established a target for all Member States to reduce NCD mortality by 25% by 2025 [4].

One of the main causes of high blood pressure is excess dietary salt intake. Excess salt intake progressively elevates blood pressure levels throughout life, which greatly increases the risks of vascular diseases [5,6] and is likely to be responsible for about half of the disease burden ascribed to high blood pressure [7]. Whilst there is still debate about the impact of salt reduction on cardiovascular disease, the totality of the evidence is convincing [8-10]. Much of the debate derives from the misinterpretation of weak research [11]. Carefully conducted overviews of the entirety of the evidence base consistently draw the same conclusions about the significant adverse effects of excess salt intake on health and the likely large health gains that can be achieved from population-wide salt reduction [12-14].

National and international organisations (including the Australian National Health and Medical Research Council [15], Health Canada [16], the American Heart Association [11], and the World Health Organization [13,17]) advocate for programs that will reduce salt intake. In addition, Member States at the World Health Assembly in Geneva in May 2013 agreed on a global target to reduce salt by 30% by 2025 [4].

The physiological requirement for salt is less than 1 gram per day [18] but, in populations where salt has been measured, average daily intakes are between 9 and 12 grams [19]. Interventions to reduce salt have demonstrated effectiveness in developed (high income) countries. Japan and Finland both implemented successful salt reduction strategies in the 1970s [20,21] and the UK’s salt reduction program, launched in 2003, has reduced average daily salt intake by about 1.5 grams (from 9.6 to 8.1 grams) in the last decade [22].

A recent review of salt reduction strategies in 32 countries [23] identified baseline data about salt intake levels and patterns as a clear prerequisite for the successful design and implementation of effective interventions. Most programs included work with the food industry to reduce the salt content of foods and meals, in parallel with campaigns to change consumer behaviour [23-25]. Voluntary or mandatory initiatives to address labelling and work to change the food environment through settings, such as schools and hospitals, were also key elements of many strategies.

Whilst there is a growing body of evidence about the likely effectiveness of different strategies to reduce salt intake, there are fewer data about their likely cost-effectiveness; and a paucity of data on salt from low and middle-income countries [26]. In Pacific Island countries, an increased reliance on processed foods is contributing to the rise in the incidence and prevalence of NCDs [27-29]. Approximately 40% of all 9.7 million Pacific Island citizens have been diagnosed with a NCD, mainly cardiovascular disease or diabetes, and/or have hypertension [30].

Surveys in a number of Pacific Islands countries, using the WHO STEP-wise approach to chronic disease risk factor surveillance (STEPS) [31], have confirmed high blood pressure to be a significant NCD risk factor [32,33]. Salt intake is almost certainly a key contributor to this problem with high sodium foods, such as bread, margarine, crackers, noodles, canned foods, butter, soy and other sauces, now staples for many households [28]. However, salt intake patterns have not been systematically and objectively evaluated to date. While the limited available information suggests high salt levels in many Pacific Island countries, more detailed country specific information is required to mount, and garner multi-sector support for, salt reduction programs [34]. For most countries it appears that a population-wide salt reduction program would potentially be one of the most cost-effective ways of improving population health [35], but the evidence to drive concerted action is mostly absent [26].

This project, funded through the Global Alliance for Chronic Disease (GACD) Hypertension Program, will help to fill these gaps in the evidence. The Program is the first initiative of its kind with the world’s largest funders of medical and health research coming together to fund research into non-communicable diseases (NCDs). Each of the fifteen research projects are being conducted through partnerships between investigators from institutions in high-income and low-and middle-income countries. A key focus of the program is implementation science and translation of evidence into policy and practice.

## Methods/Design

The study will employ a before-after design. Baseline (2012) and follow-up surveys (2015) will be conducted either side of a multi-pronged, cross-sectoral intervention that seeks to reduce population salt intake in Fiji and Samoa over two years. The study will be conducted in three phases and will use both quantitative and qualitative research methods. Broadly, these three phases constitute:

*– Phase I- Baseline assessments* comprising population surveys of salt intake, surveys of the salt content of foods in shops, participatory community research to understand stakeholder views, and an audit of current practice.

*– Phase II – Intervention development and implementation* using the baseline information to formulate a comprehensive policy response and action plan to reduce population salt intake.

*– Phase III – Evaluation of impact* through follow-up community surveys, surveys of foods in shops and a cost-effectiveness analysis.

### Study objectives

The overall study objective is to assess the effect of a multi-pronged, cross-sectoral intervention program to reduce population salt intake in both Fiji and Samoa. Secondary objectives include determining the effects of the salt reduction program on population knowledge, attitudes and behaviour by comparing mean population levels at baseline and follow-up and determining the effects of the salt reduction program on average levels of sodium in foods based upon food composition surveys that collect data from food labels at baseline and follow-up. The project will also consider the effects of the salt reduction intervention on dietary salt by examining changes in diet based on the Food Frequency Questionnaires (FFQs) administered at baseline and follow-up in each country. The relationship between salt intake measured by 24-hour urine and salt intake estimated from spot urine will also be explored.

### Outcomes

#### Primary

The primary outcome of the study will be the change in 24-hour urinary salt level in the overall study population (in each country). If a positive impact is demonstrated, then a cost-effectiveness assessment will also be undertaken.

#### Secondary outcomes

The main secondary outcomes will be changes in knowledge levels and current practices relating to salt and changes in the mean concentration of sodium in foods.

Additional outcomes will include changes in the composition of diet based on the FFQs and understanding of the relationship between salt intake measured by spot and 24-hour urine in each country. The latter is important as, if it proves possible to use spot urines to estimate 24-hour urinary sodium excretion, in future this would provide a more feasible and practical alternative to measuring salt intake for countries.

#### Sampling strategy

The study population will be adults aged 18–69 years in both countries. Baseline salt assessment and questions on knowledge, attitudes and behaviours related to salt (KAB) will be integrated into the WHO STEPS survey in Samoa and the STEPS sampling framework will be used in Fiji. The framework uses a stratified (province or division) three-stage (enumeration area, household and individual) cluster random sampling process using probability proportional to size to obtain representative samples for the STEPS survey in each country based on the statistical regions identified in recent Censuses [36-38]. Sub-samples (e.g. every fifth person, so as to recruit 250 men and 250 women) will then be selected to collect 24-hour and spot urines.

#### Recruitment

Before beginning recruitment, permission will be sought from the local village chief to enter the village or enumeration area to carry out the work. Study enrolment and data collection will be conducted face-to-face in English (which is spoken by more than 90% of people in Fiji and Samoa) by trained health research staff. The research assistants will assist participants by orally translating written material into the local language, if needed. Participant information sheets and consent forms will be available in Hindi and Fijian (for Fiji) and Samoan. Each consented participant will be assigned a unique identification number so that survey results can be kept separate from personal details to ensure data confidentiality.

#### Data collection and management

Interviewer administered questionnaires will be used to obtain demographic and risk factor related information, including knowledge, awareness and practices related to salt from all participants. In addition, dietary intake will be assessed by trained research assistants using food frequency questionnaires (FFQs) on the sub-sample. The FFQ has been previously used to measure population salt intake in Fiji [39]. It will be adapted for Samoa, informed by the list of foods collected during the STEPS survey. Spot and 24-hour urine samples will be collected for each participant in both countries in accordance with established protocols [40]. All methods will be piloted as part of the training processes in each country.

#### Sample size

The sample size for each survey has been calculated to provide precise estimates of the average level of salt separately in adult Samoans and Fijians, based on the 24-hour urine collections. Five hundred participants recruited in both Fiji and Samoa will allow reporting of the mean level of sodium intake to within +/− 4.2 mmol with 95% confidence for each country. This estimate assumes a mean intake of 160 mmol sodium per day with a standard deviation of 48 mmol (1 gram of salt = 17.1 mmol sodium). This sample size will also enable the mean level of sodium to be reported to within +/− 5.85 mmol, for men and women and for rural and urban areas (where the sample is effectively divided in two with a sample size of 250 in each group), and to within +/− 8.42 mmol for subgroups such as older vs. younger men or rural versus urban women (where there will be a sample size of about 125 for each group). There will also be 80% power (alpha = 0.05) to detect approximately 8.5 mmol (0.5 gram salt) or greater differences between the average sodium levels between baseline and follow-up for each country.

#### Analysis methods

The spot and the 24-hour urine samples from each individual will be analysed separately for sodium, potassium and creatinine. All analyses will be done by local laboratories in Samoa and Fiji using the ion selective electrode method for sodium and potassium analysis and the buffered kinetic Jaffe reaction without de-proteinisation for urine creatinine assay. Age and sex specific estimates obtained from the survey will be weighted based on population data from the most recent census. Descriptive statistics will be reported for anthropometrics, consumer awareness and urinary sodium levels. Results will be reported by sex age groups and evidence for differences between subgroups examined using t-tests.

### Surveys of the salt content of foods for sale in shops

#### Sampling strategy

The salt levels in the foods that contribute most to salt in the diet (identified through the FFQs) will be monitored according to published protocols [41] with the largest stores in the main urban centres of Fiji (Suva) and Samoa (Apia) included in the surveys. Additional chemical analysis of the sodium content of the main meals eaten in and out of the home will be undertaken by national government laboratories in each country.

#### Data collection and management

The food composition data will be collected primarily from printed information on the front and back of the product labels, including the nutrition information panel. The minimum data fields collected for each product will be country of origin, brand/manufacturer, product name, food category, serving size and sodium content per 100 g and/or per serving. Direct chemical analysis of the sodium content of a sub-sample of products will be undertaken to assess the accuracy of the sodium content listed on the nutrition labels. Direct chemical analysis will also be used to obtain sodium content data for products and meals that have been identified through the FFQ as contributing significantly to salt in the diet and that are unpackaged or do not display a nutrient declaration.

#### Analysis methods

The primary analysis will compare the mean concentration of sodium in foods before and after the salt reduction intervention has been implemented. Subsidiary analyses will explore changes in sodium levels in different food types and for the foods marketed by different manufacturers.

### Development of the salt reduction program

The development of the salt reduction program will be under-pinned by participatory community research [42] to obtain a comprehensive understanding of consumer and stakeholder opinions in relation to the most effective mechanisms for reducing salt intake. Stakeholder consultation will be conducted in both Fiji and Samoa and, while it is anticipated to be considerable overlap in the findings, the different structural social and cultural factors in each country will be carefully considered.

#### Participatory community research

Focus groups of up to two hours’ duration will be held with key stakeholders using a trained local professional research person and a semi-structured interview question format. The sessions will focus on assessment of potential barriers and opportunities for change in relation to reducing salt intake or salt levels in foods, and the likely impact, feasibility and acceptability of different possible interventions.

Representatives of the food industry (manufacturers, importers, caterers, ingredients suppliers), government (Ministries of Health, Transport, Commerce and Industry, Finance and Women and Culture), consumers, NGOs and Church Groups and international organisations, will be invited to participate in approximately 10 focus groups (of 8–10 participants ) in each country, allowing for a broad range of opinion. Purposive snowball sampling [43] will be used to identify stakeholders in each country with existing organisations, such as women’s groups and church groups, used to identify additional potential participants. Separate focus groups will be conducted for stakeholders from different sectors (government, industry, community). The assessment of the potential interventions will be guided by background information about existing national and regional commitments, and relevant evidence on policy options. The focus group sessions will be recorded and subsequently transcribed verbatim.

Focus group transcripts will be coded and organized according to key themes with analysis that seeks to elucidate barriers and opportunities for change and the feasibility and acceptability of different interventions. The findings will be explored overall and separately for the consumer, government and industry groups. At the completion of the analysis, the possible interventions will be ranked on the basis of potential impact, feasibility and acceptability. The key secondary analysis will be a detailed assessment of the main barriers and opportunities for change.

#### Salt reduction intervention

While the nature of the salt reduction intervention strategies will be informed by the outcome of the stakeholder consultation and other baseline data collected during the project, it is likely that the program will include core components and approaches employed elsewhere. In addition, the strategies will reinforce existing national programs being implemented by the Ministry of Health and key agencies in each country. Core components of the programs will include: (i) the food industry (including caterers, bakers, importers, processors and retailers) with efforts directed towards reducing average salt levels in locally manufactured and imported foods towards agreed targets for salt levels in foods, work to facilitate the catering and food service sectors to provide low salt meals and initiatives related to labelling of foods in stores and food service outlets; (ii) consumers through targeted and sustained campaigns to change awareness and behaviours related to salt with a focus on children and using culturally appropriate communication channels including church leaders and women’s groups; and (iii) settings such as schools, hospitals and the workplace through the establishment of standards and educational campaigns.

### Cost-effectiveness analysis

Incremental cost-effectiveness analysis will be undertaken to determine whether the intervention strategy in each country represents ‘value-for-money’ measured against current practice. It will address issues of both technical efficiency (‘how to do it’) through assessment of key design features of the intervention strategy and the associated cost drivers, and allocative efficiency (‘what to do’) through the modelling of longer term consequences and cost offsets. The analyses will be conducted from a societal perspective for the 2013 reference year. Pathway analysis (‘who does what to whom, when and where and how often’) will be used to specify resources associated with the interventions. Detailed documentation of all activities undertaken, and their associated resource costs, will be conducted throughout the study duration to facilitate comprehensive costing of interventions (excluding and including measurements).

Economic modelling will be used to extend the target population, time horizon and decision context for the cost-effectiveness analysis. The incremental change in salt consumption, as a result of the intervention strategy, will be converted to savings in Disability-Adjusted Life Years (DALY) over the lifetime of the national cohort (Fiji and Samoa separately), with the resulting incremental cost-effectiveness ratios expressed as cost per DALY saved. Standard discounting will be applied to both costs and outcomes. Simulation-modelling using the @RISK software package will be employed to calculate 95% uncertainty intervals (around the median) about the efficacy and cost estimates.

### Ethics and dissemination

Ethics approval for this study has been obtained from the Human Research Ethics Committee at the University of Sydney and Deakin University in Australia and from the local ethics committees in Fiji (Fiji National Ethics Review Committee) and Samoa (Ministry of Health).

Results will be presented and made available to MOH staff in each country, published in a high quality peer-reviewed journal and disseminated widely through conferences and events, using the George Institute, C-POND and Global Alliance for Chronic Disease stakeholder networks.

## Discussion

The potential for salt reduction to reduce the burden of NCDs has now been endorsed internationally [44], with projections in recent studies that interventions would save millions of lives a year and be cost-saving to governments in the long run [35,45]. Samoa and Fiji both have relatively strong approaches to NCD prevention and have already highlighted salt reduction as a priority. Existing data on salt intake from Pacific Island countries are, however, sparse and this study will directly benefit Fiji and Samoa and be a major step forward in enabling the development and implementation of local salt reduction programs [46]. Both countries have identified the need for accurate information on current salt intake and sources of sodium in the diet to inform their plans for interventions. As such, there is a high likelihood that this project will translate into practical interventions with the potential to save lives and avert disease burden in the short term.

Key strengths include important new scientific data and capacity building in relation to monitoring and surveillance, intervention implementation and cost-effectiveness evaluation of salt reduction interventions in Fiji and Samoa. The project will use the gold standard method of 24-hour urine collection to monitor salt intake. Integrating the monitoring into the WHO STEPS program will ensure the collection of high quality data on a representative population sample and be crucial in ensuring the future sustainability of the salt monitoring as it is recommended that STEPs NCD risk factor surveys are repeated every five years. The participatory nature of the approach involving a diverse range of stakeholders will result in important contextual information which should improve the effectiveness of the intervention strategies. In both countries, the project will involve government throughout the process of developing and implementing policiesx and interventions, which should facilitate knowledge translation and ensure that salt reduction remains on the government agenda. The sample size will ensure adequate power to detect a small but clinically significant change in salt intake over a relatively short period.

The study has a before and after design with no control group. However, whilst randomised control trials are the gold standard, it is not always possible to replicate them in real life settings and so the design described here is more appropriate. Differences in terms of governance (principal research staff employed in research organisation in Fiji and in the Government Health Department in Samoa), and interventions (building on the on-going strategy in Fiji and developing a new strategy in Samoa) will also need to be taken into account when considering the effectiveness and constitute a key strength of the study. Process evaluations will enable us to make judgements about which interventions are more likely to be effective and help to identify the more feasible and practical approaches for salt reduction, particularly for low and middle-income countries with limited resources. Important new evidence on the appropriateness of spot urines to estimate 24-hour urinary sodium excretion in the Pacific Islands will be another key outcome of the study. All of these findings will be used to inform WHO guidance around sodium currently being developed and as such will have an immediate impact on international policy and practice. This will increase the potential for all countries to achieve the new global targets for salt reduction and as such have a major impact on NCDs around the world.

## Abbreviations

C-POND: Pacific Research Centre for the Prevention and Control of Obesity and Non-Communicable Diseases; DALY: Disability- Adjusted Life Years; FFQ: Food Frequency Questionnaire; KAB: Knowledge Attitudes and Behaviour Survey; NCD: Non-communicable disease; STEPS: WHO-STEP-wise approach to chronic disease risk factor surveillance; WHO: World Health Organization.

## Competing interests

JW is Director of the WHO Collaborating Centre on Population Salt Reduction. BN is Chairman of the Australian Division of World Action on Salt and Health. No other authors have any competing interests to declare.

## Author’s contributions

JW, WS, MM, SV, JS, BN, MW, CB and ED contributed to the study design. FB and MW advised on the statistical analysis plan. JW and SD drafted the manuscript with input and review from all other authors. All authors read and approved the final manuscript.

## Pre-publication history

The pre-publication history for this paper can be accessed here:

http://www.biomedcentral.com/1471-2458/14/107/prepub

## Research partners

This project is being conducted by The George Institute for Global Health in partnership with Deakin University in Australia and the Pacific Research Centre for the Prevention and Control of Obesity and Non-Communicable Diseases (C-POND), a partnership between Deakin University and Fiji National University in Suva, Fiji. Local partners include the Fiji National Food and Nutrition Centre and the Samoan Government (Ministry of Health) and the World Health Organization’s South Pacific Office in Suva, Fiji.
